# The importance of instrumental assessment in disorders of consciousness: a comparison between American, European, and UK International recommendations

**DOI:** 10.1186/s13054-022-04119-5

**Published:** 2022-08-10

**Authors:** F. G. Magnani, F. Barbadoro, M. Cacciatore, M. Leonardi

**Affiliations:** UOC Neurologia, Salute Pubblica Disabilità - Coma Research Centre, Fondazione IRCCS Istituto Neurologico Carlo Besta, Milan, Italy

**Keywords:** Disorders of consciousness, Vegetative state, Unresponsive wakefulness syndrome, Minimally conscious state, International guidelines, Functional imaging, Electrophysiology, Diagnosis, Prognosis

## Abstract

The use of instrumental tools for improving both the diagnostic accuracy and the prognostic soundness in patients with disorders of consciousness (DOC) plays an important role. However, the most recent international guidelines on DOC published by the American and the European Academies of Neurology and by the UK Royal College of Physicians contain heterogeneous recommendations on the implementation of these techniques in the clinical routine for both diagnosis and prognosis. With the present work, starting from the comparison of the DOC guidelines’ recommendations, we look for possible explanations behind such discrepancies considering the adopted methodologies and the reference health systems that could have affected the guidelines’ perspectives. We made a provocative argument about the need to find the most appropriate common methodology to retrieve and grade the evidence, increase the meta-analytic studies, and reduce the health policies that influence on the guidelines development that, in turn, should inform the health policies with the strongest scientific evidence.

**Supplementary Information:**

The online version contains supplementary material available at 10.1186/s13054-022-04119-5.

## Introduction

After acquired brain injuries, patients may present with a prolonged alteration of consciousness (i.e., disorders of consciousness; DOC) along a continuum from coma to severe disability. Patients in a coma state are characterized by the absence of the sleep–wake cycle, while patients emerging from this condition may present with different levels of responsiveness. Specifically, when patients are awake but not responsive, they are diagnosed with vegetative state (VS, also known as unresponsive wakefulness syndrome; UWS [1]); patients manifesting reproducible, even if not consistent, evidence of self- and/or environmental awareness are diagnosed with minimally conscious state (MCS) [2]. To date, the clinical distinction between VS/UWS and MCS relies on behavioral assessment referring to the Coma Recovery Scale—Revised (CRS-R) as the gold standard [3]. Although the CRS-R is able to reduce the misdiagnosis rate [4], the diagnostic uncertainty endures if the patient does not manifest any kind of behavioral responses.

A growing number of studies highlight the possibility to assess the patients’ level of consciousness with instrumental tools in the absence of any behavioral signs [5–8]. One of the first pieces of evidence derives from the study by Owen et al. [9] which described a patient who, while fulfilling the clinical criteria for VS/UWS, showed brain activations as healthy subjects in motor imagery tasks [9], thus suggesting a change in diagnosis. This result paved the way for the use of complementary tools to assess the patients’ level of consciousness. Indeed, paradigms using functional magnetic resonance imaging (fMRI), electroencephalography (EEG), diffusion tensor imaging (DTI), positron emission tomography (PET), and combined techniques (e.g., transcranial magnetic stimulation and EEG; TMS-EEG) all show the possibility to detect something not detectable with behavioral assessment [9–13], thus potentially improving the diagnostic accuracy in DOC [14]. Furthermore, evidence about the prognostic value of these techniques is enlarging as well [15–17]. Most of the prognostic studies on DOC focused on the role of the default mode network adopting resting-state fMRI (rs-fMRI) paradigms [18, 19], although the use of other techniques seems to be promising as well (see for instance [20–22]). Notwithstanding this encouraging evidence, we are still far from having suitable and reliable protocols able to accurately predict DOC patients’ outcomes, which is one of the pivotal aims of the international research agenda.

Even the most recent guidelines on DOC, namely the American Academy of Neurology (AAN) [23], the European Academy of Neurology (EAN) [24], and the UK Royal College of Physicians (RCP) [25], differ in considering the role of the instrumental tools for improving the diagnosis and prognosis of DOC patients. Specifically, they adopted different and already debated [26, 27] positions concerning the use of instrumental tools in the clinical routine with DOC patients (Table 1). Indeed, the AAN guidelines (weakly) recommend the use of instrumental tools including functional imaging and electrophysiological techniques when there is ambiguity in behavioral assessment or when there are confounders to a valid diagnosis. Furthermore, they moderately recommend the use of SPECT and MRI for prognostic reasons. The EAN guidelines, instead, provide a strong recommendation for the use of the standard EEG visual analysis, while they weakly recommend other neuroimaging and neurophysiological techniques for diagnostic purposes. They do not provide any prognostic recommendation. Finally, the UK RCP guidelines exclude either the diagnostic or prognostic use of instrumental tools in the clinical routine, deeming it necessary only if the results will alter clinical management, and always declaring to consider the patient’s best interest [25].

**Table 1:** Diagnostic and prognostic use of instrumental tools recommendations in patients with disorders of consciousness

Guideline	Recommendation no.	Recommendation	Prognosis/diagnosis	Level of recommendations
*AAN*	2e	In situation where there is continued ambiguity regarding evidence of conscious awareness despite serial neurobehavioral assessments, or where confounders to a valid clinical diagnostic assessment are identified, clinicians may use multimodal evaluations incorporating specialized functional imaging or electrophysiologic studies to assess for evidence of awareness not identified on neurobehavioral assessment that might prompt consideration of an alternate diagnosis	Diagnosis	C (weak)
	5	In post-traumatic VS/UWS patients, clinicians [..] may assess for the presence of P300 at 2–3 months post-injury or assess EEG reactivity at 2–3 months post-injury to assist in prognostication regarding 12-month recovery of consciousness for patients in traumatic VS/UWS	Prognosis	C (weak)
		In post-traumatic VS/UWS patients, clinicians should perform MRI 6–8 weeks post-injury to assess for corpus callosal lesions, dorsolateral upper brainstem injury, or corona radiata injury in order to assist in prognostication regarding remaining in PVS at 12 months for patients in traumatic VS/UWS	Prognosis	B (moderate)
		In post-traumatic VS/UWS patients, clinicians should perform a SPECT scan 1–2 months post-injury to assist in prognostication regarding 12-month recovery of consciousness and degree of disability/recovery for patients in traumatic VS/UWS	Prognosis	B (moderate)
		In post-traumatic VS/UWS patients, clinicians may assess for the presence of higher-level activation of the auditory association cortex using BOLD fMRI in response to a familiar voice speaking the patient’s name to assist in prognostication regarding 12-month (post-scan) recovery of consciousness for patients in traumatic VS/UWS 1–60 months post-injury	Prognosis	C (weak)
	6	In non-traumatic post-anoxic VS/UWS patients, clinicians [..] may assess SEPs to assist in prognostication regarding recovery of consciousness at 24 months	Prognosis	C (weak)
*EAN*	*Functional neuroimaging*			
	*PICO 1*	Resting-state fluorodeoxyglucose (FDG) PET may be considered as part of multimodal assessment in unresponsive patients	Diagnosis	Low evidence, weak recommendation
	*PICO 2*	If a standard clinical (structural) MRI is indicated, it is suggested that a resting-state fMRI sequence is added as part of multimodal assessment	Diagnosis	Low evidence, weak recommendation
	*PICO 3*	It is suggested to add a resting-state fMRI sequence as part of multimodal assessment whenever a standard (structural) MRI is indicated; however, the default mode network is just one of several resting-state fMRI networks that may be used to complement the behavioral assessment in patients with DOC	Diagnosis	Low evidence, weak recommendation
	*PICO 4*	It is suggested that passive fMRI paradigms be used within research protocols	Diagnosis	Low evidence, weak recommendation
	*PICO 5*	It is suggested that active fMRI paradigms should be considered as part of multimodal assessment in patients without command following at the bedside	Diagnosis	Moderate evidence, weak recommendation
	*PICO 6*	It is therefore suggested that salient stimuli should be used for examination of DOC patients by fMRI	Diagnosis	Very low evidence, weak recommendation
	*EEG-based techniques, including TMS-EEG and evoked potentials*			
	*PICO 1*	Visual analysis of clinical standard EEG seems to detect patients with preserved consciousness with high specificity but low sensitivity	Diagnosis	Low evidence, strong recommendation
	*PICO 2*	Non-visual (i.e. numerical) analysis of standard EEG cannot yet be recommended for the differentiation between VS/UWS and MCS	Diagnosis	Very low evidence, weak recommendation
	*PICO 3*	It is suggested that sleep EEG be used for the differentiation between VS/UWS and MCS as a part of multimodal assessment	Diagnosis	Low evidence, weak recommendation
	*PICO 4*	It is suggested that quantitative analysis of high-density EEG be considered for the differentiation between VS/UWS and MCS as part of multimodal assessment	Diagnosis	Moderate evidence, weak recommendation
	*PICO 5*	Cognitive evoked potentials for the differentiation between VS/UWS and MCS might be considered as part of multimodal assessment	Diagnosis	Low evidence, weak recommendation
	*PICO 6*	It is suggested that TMS-EEG should be considered for the differentiation between VS/UWS and MCS as part of multimodal assessment	Diagnosis	Low evidence, weak recommendation
*RCP*	*2.7*	It is not yet clear whether more sophisticated electrophysiology and brain imaging techniques (e.g., fMRI, PET, DTI) have any diagnostic or prognostic utility over and above expert clinical and behavioral assessment	Diagnosis and prognosis	E1/2
		(a) They do not form part of the standard assessment battery for PDOC at the current time, nor do they represent a ‘practicable step’ required by s.1(3) MCA 2005 to support a person’s capacity to make relevant decisions		
		(b) Further work is required to understand the relationship between these and the formal clinical evaluation tests		
		(c) In the meantime, they should be only applied in the context of a registered research program and in conjunction with formal clinical evaluation as described in recommendation 2.4		

The recommendation(s) for each guideline are displayed along with reference to their diagnostic or prognostic utility, and their level (last column)

VS = vegetative state; UWS = unresponsive wakefulness syndrome; EEG = electroencephalogram; MRI = magnetic resonance imaging; PVS = persistent vegetative state; SPECT = single-photon emission computerized tomography; SEp = somatosensory evoked potential; fMRI = functional magnetic resonance imaging; PET = positron emission tomography; DOC = disorders of consciousness; MCS = minimally conscious state; TMS = transcranial magnetic stimulation; DTI = diffusion tensor imaging; PDOC = prolonged disorders of consciousness; MCA = mental capacity act

However, the reasons behind these discrepancies are far to be clear. Thus, the present work aimed at comparing the guidelines’ recommendations on the use of instrumental tools for improving the diagnosis and prognosis of DOC. Specifically, starting from the guidelines’ recommendations on the adoption of instrumental tools in DOC, we look for possible explanations of such discrepancies considering (i) the adopted methodologies to produce the recommendations, and (ii) the National Health System as a context where the recommendations have to be applied.

## The role of the adopted methodologies in explaining the recommendations discrepancies

Some discrepancies are evident when comparing the recommendations on the use of instrumental tools for improving diagnosis and prognosis in DOC of the three most recent guidelines (see Tables 1 and 2 for an overview).

**Table 2:** Main features of guidelines on DOC

Guideline	Year	Systematic review availability	Grading system	Inclusion criteria		No. of recommendations		Tools specification		Etiology specification		Recommendations level		NHS
				Population features	Methodological features	*Dx*	*Px*	*Dx*	*Px*	*Dx*	*Px*	*Dx*	*Px*	
AAN	2018	Yes	GRADE	Yes	Yes	1	5	No	Yes	No	Yes	Weak	Weak (n = 3); Moderate (n = 2)	Private
EAN	2020	Yes	GRADE	Yes	Yes	12	0	Yes	Not available	No	Not available	Weak (n = 11); Strong (n = 1)	Not available	Public/Private
RCP	2020	No	NSF for long-term conditions	Yes	No	1	1	No	No	No	No	Weak	Weak	Public

The table depicts for each guideline (i) the year of publication, (ii) the methodological features of each guideline including the adopted evidence grading system and inclusion criteria, (iii) the total amount of recommendations for both diagnosis (dx) and prognosis (px), (iv) recommendations’ specifications concerning tools and etiology, (v) recommendations’ level for both dx and px with the number of recommendations falling within a specific recommendation’ level in the brackets, and (vi) the reference health system

AAN = American Academy of Neurology; EAN = European Academy of Neurology; RCP = Royal College of Physicians; NSF = National Service Framework; GRADE = Grading of Recommendations Assessment, Development and Evaluation; Dx = Diagnosis; Px = Prognosis; NHS = National Health System

Although all the recommendations were derived from a systematic review, we were able to retrieve the search strategies and results of both the AAN and EAN review process, while the UK RCP did not make anywhere public either the search strategy or the search results, thus making impossible a direct comparison between the evidence each guideline relied on to derive the recommendations (see Additional file 1 for a direct comparison between the evidence included by the AAN and EAN).

Furthermore, AAN, EAN, and UK RCP relied on very different methodologies to grade evidence and, consequently, to build recommendations. Specifically, the RCP guidelines are based on the National Service Framework for long-term conditions typology [28] which differs from the traditional evidence evaluation system used by both AAN and EAN (i.e., Grading of Recommendations Assessment, Development and Evaluation—GRADE approach [29–31]) in considering also the opinions and the experience of service users, caregivers, families, and professionals [32]. This difference poses an issue in comparing the recommendations between the three guidelines as the RCP may have included some evidence deriving from users, families, and caregivers that have not been considered by AAN and EAN. Indeed, the RCP recommendation concerning the adoption of complementary instrumental tools for diagnosis and prognosis reaches the E1/2 level of recommendation (see Table 1), meaning that it derives from consultation or consensus processes rather than formal research designs [25].

Moreover, the RCP guidelines adopted broader inclusion criteria than those adopted by the AAN and EAN. Specifically, they considered evidence (i) concerning patients over 16, (ii) having a prolonged DOC (> 4 weeks), and (iii) set date constraints during the review process according to the use of the specific terminology (i.e., the term “Minimally Conscious State” started to be used from 2002 [2]). On the contrary, AAN and EAN adopted more specific inclusion criteria not limited to clinical population features (i.e., for both AAN and EAN age > 18; for AAN time from the acute event ≥ 28 days) and publication dates (i.e., for AAN from 1950 to February 2017; for EAN from 2002 to December 31, 2018). Indeed, they also considered some methodological features driving evidence’s eligibility to be based on to develop recommendations. The AAN included only the studies enrolling at least 20 patients and only studies not relying on a comparison between DOC patients and healthy controls for diagnostic recommendations, as there is no “*any diagnostic uncertainty in this comparison*” (Additional file 1 of [23], p. 5). The EAN, instead, included only studies with a sample size > 3 displaying data at a single-subjects level, without considering studies reporting data already described in other works by the same authors/institutions; they also did not consider prognostic studies that do not employ consciousness paradigms [24]. Taken together, the above-mentioned discrepancies among the three guidelines might suggest that the adoption of heterogeneous inclusion criteria during the review process brought some differences in the recommendations’ contents. It is not surprising that different data lead to different results.

Notwithstanding the above-mentioned discrepancies, the three guidelines are consistent in one important respect as most of the evidence for the use of instrumental tools in DOC patients falls in the low evaluation category with a consequent weakness of the recommendations (see Table 2). The only exception is the EAN guidelines which strongly recommend the use of visual analysis of clinical standard EEG for diagnostic purposes. Specifically, despite the lowness of the evidence, the visual analysis of standard EEG is strongly recommended by the EAN due to its high specificity. Similarly, the AAN moderately recommends performing MRI and SPECT around 1–2 months from acute event to support prognosis in post-traumatic patients who are in VS/UWS.

However, none of the guidelines provides a practical framework for clinicians who are the persons facing the ‘reliability dilemma’ of the assessment procedures to adopt for reducing the misdiagnosis rate and improving the prognostic soundness. In other words, the guidelines do not allow to infer which instrumental tools have to be applied case-by-case within a personalized medicine approach. For the sake of clarity, the AAN provides different recommendations (only for prognosis) depending on patients’ etiology limiting them, however, to post-acute stages (> 28 days from the acute event), overlooking some results deriving from acute patients. Conversely, the EAN, while not considering time limits from the acute event, does not provide suggestions for a case-by-case selection of the most appropriate instrumental tools as well (and not at all for prognostication).

Overall, although the AAN, EAN, and UK RCP acknowledged the lack of high level of evidence to support the use of instrumental tools in the clinical routine with DOC patients, they adopted different positions: A more conservative one is perceivable within the RCP guidelines, while a more positive attitude is detectable within AAN and EAN guidelines.

## The role of different national health systems and structures in explaining the recommendations discrepancies

Besides the above-mentioned methodological reasons, AAN, EAN, and UK RCP might have other specific reasons to be more or less restrictive on the use of instrumental tools in the clinical routine with DOC patients. It is indeed important to consider that some differences exist in healthcare systems and structures among different countries, as already highlighted by Wade et al. [27] in their reply to Scolding et al. [26]. Indeed, the authors claimed the need to place the guidelines on DOC in the context of the specific healthcare systems.

In our view, the main difference is the degree of private/public funding in the healthcare system (Table 2). It is thus not unreasonable to see a more conservative position characterizing public health systems using public funds, given that some of the instrumental tools are costly. Let’s think for instance fMRI and PET examinations: Given the still-high uncertainty of their diagnostic and prognostic value for DOC patients and their associated costs, it is not surprising that public health systems, like the UK one, keep a cautious position on their standard use in the absence of strong evidence. On the contrary, the more positive attitude of the AAN concerning the use of instrumental tools (including neuroimaging) with DOC patients may relate to a different payment system in the USA that reflects the general tendency in performing imaging examinations for all clinical conditions. Indeed, a recent JAMA editorial on the costs of the US healthcare system [33] identified the imaging volume (i.e., number of performed examinations) and prices as two of the drivers of the US healthcare costs compared to other high-income countries. The author highlighted that the US “*performs many more CT scans than any other country and is the second highest user of MRI worldwide*” ([33]; p. 983). It is not surprising, therefore, to find a sort of conformity between the attitude of the national guidelines for DOC and the national practice. The same positive attitude toward the use of instrumental tools with DOC patients is perceivable within the EAN guidelines, despite Europe being characterized by mixed public/private health systems when considering all EU member states. Although the healthcare system’s structure could have played a role in orienting the guidelines’ attitude concerning the use of instrumental tools in clinical routine with DOC, it remains only a hypothesis.

The instrumental tools’ availability could be another important determinant possibly driving the recommendations and explaining their differences. The importance to have adequate infrastructures and technologies has been already listed, along with financial resources, as one of the main conditions under institutional responsibility playing a role in operationalizing guidelines recommendations [34]. We cannot exclude that this same determinant played a role in orienting the guidelines’ attitude. Indeed, for instance, the UK MRI report commissioned by the UK Clinical Imaging Board in 2017 [35] stated that the UK’s MRI capacity was 6.1 MRI systems per million people, thus much lower compared to other countries such as the USA which counted 38.1 MRI systems per million people. Therefore, one may speculate that a system having all the necessary infrastructures encourages their use more than a system having less infrastructures’ availability. However, we do not have enough evidence to confirm this hypothesis, thus remaining speculative. Indeed, some members of the UK multidisciplinary guideline development group highlighted that “*many of [UK] recommendations require more resources than are available[..]*” (pp. 7–8; [27]).

Considering UK, US, and EU healthcare systems’ differences, it is difficult to conceive how RCP, AAN, and EAN would align their tendencies toward the use of instrumental tools in the clinical routine with DOC patients. Would the RCP agree to a more comprehensive (and costly) diagnostic approach, despite low evidence in showing major gains for the patients? Or would the AAN and EAN dilute their recommendations on the use of instrumental tools to assimilate to the UK public health perspective?

## Discussion

The use of instrumental tools in the clinical routine of DOC patients is still debated. Even the most recent guidelines developed by the AAN [23], EAN [24], and the UK RCP [25] differ in recommending the use of instrumental tools for both diagnosis and prognosis. Specifically, while the AAN [23] and EAN [24] guidelines suggest the diagnostic use of neuroimaging and electrophysiological techniques, albeit the weakness of the recommendations, the UK RCP guidelines [25] reject their adoption in the clinical routine and recommend them only in some specific cases (e.g., patients’ best interest). A more uncertain scenario appears when considering the instrumental tools recommendations for prognostic reasons: While the AAN guidelines provide different recommendations depending on the patient’s etiology, the UK RCP guidelines discourage their adoption to date, and the EAN does not provide any recommendation concerning the prognostic use of the instrumental tools. The reasons behind such discrepancies could derive mainly from the adoption of different methodologies to retrieve and grade the existing evidence, that inevitably lead to the inclusion of some evidence over others, increasing the risk to overlook some important results, and thus missing solid evidence-based roots. Overall, there is an urgent need to align the methodologies within the international scientific community when building guidelines for clinical practices, by defining, for instance, the more reasonable inclusion/exclusion criteria depending on the topic, and by sharing international validated systems for grading the evidence.

There are still many unanswered questions concerning patients with DOC, especially on the prognostic determinants, as confirmed by the lack of any recommendations on the prognostic use of instrumental tools in the EAN guidelines and the little evidence provided by the AAN ones. These data disclose the paucity of longitudinal studies on DOC patients analyzing the prognostic value of different instrumental tools, as well as the difficulty in adopting specific techniques, such as the fMRI, in very frail patients. This difficulty strongly depends also on the availability of the techniques and on the possibility to move patients according to their medical stability. It is thus very important to start by pooling together the efforts already done by different laboratories, clinical centers, and institutions in exploring both the prognostic and the diagnostic value of instrumental tools by means of meta-analytic studies (see for instance [17]) that should become the solid base to develop national and international guidelines.

Besides the methodological difference among the guidelines, we hypothesized the role of the health systems and structures in orienting the guidelines attitudes toward the adoption of specific instrumental tools in the clinical routine with DOC. Although stakeholders’ involvement should not represent a problem with guidelines development, there might be issues related to political, economic, and cultural reasons that might affect editorial independence, and this is not new (see for instance [36] on a different topic). However, our hypothesis remains speculative as we cannot provide empirical evidence to prove that health systems’ peculiarities have influenced the guidelines development. Even assuming such an influence, several methodological differences remain and should be resolved first. Nevertheless, the health systems and structures surely affect the operationalization of the guidelines recommendations as well pointed out by Farisco et al. [34]. Indeed, the authors stated that the unavailability of some instrumental tools, insufficient economic resources, and the lack of expertise in using the instrumental tools in some contexts greatly mine the reliability and the practical meaning of some recommendations concerning the diagnostic and prognostic value of the instrumental tools in the clinical routine with DOC. Moreover, we think that the operationalization of the recommendations is made even more difficult by the different conceptual structure adopted by each guideline. Indeed, the use of instrumental tools should be differently grounded on DOC patients’ clinical stage and condition. For example, EEG, TMS-EEG, PET, and fMRI seem to be useful to detect consciousness in behaviorally unresponsive patients [13, 37–39]. Conversely, the use of these techniques seems to be poorly useful with patients who already show at least minimal behavioral responsiveness [37, 39]. Nevertheless, the three international guidelines do not clarify when specific instrumental tools should be adopted in clinical routine case-by-case. To fill this gap, previous works provided decision trees taking into account the patients’ stage, their behavioral responsiveness (i.e., diagnosis), the clinical status, confounding factors [39, 40], and even the costs associated with the use of different instrumental tools [41]. Notwithstanding these efforts, it remains to be determined a shared algorithm that can consider the peculiarities of each National Health System, the different national care pathways, legislations, economics, cultural, and moral attitudes toward very frail patients, along with the availability of instrumental tools in both acute and post-acute care settings.

Of course, the common underlying problem in having reliable and strong recommendations for adopting the instrumental tools in the clinical routine with DOC lies in the level of evidence produced by the literature which, to date, is low, thus preventing recommendations being made at a high level. However, the scientific community has to deal with the few and heterogeneous available evidence, finding the best methodological way to put them together. Discussing the level of the evidence relying on to build recommendations is out of the scope of the present work. We aimed, instead, to shed light on possible reasons driving the heterogeneous recommendations development among different guidelines.

## Conclusions

An accurate diagnosis of DOC is essential for patients' care and therapeutic management; the ability to detect and differentiate consciousness levels, thus the capacity to define a patient as conscious or unconscious has, indeed, a great impact on the rehabilitation process and even on end-of-life decisions. The use of instrumental tools for improving diagnostic and prognostic soundness should be encouraged in an evidence-based manner. Although the most recent international guidelines on DOC provide some recommendations concerning the use of instrumental tools for both diagnosis and prognosis, their methodological heterogeneity in retrieving and grading the evidence does not allow to draw firm conclusions.

For this reason, it is necessary to align the methodology behind international and national recommendations’ development and to find the best way to put together all the available data despite their low level of evidence.

## Supplementary Information

**Additional file 1:** Comparison between American and European Academies of Neurology eligible studies.

## Data Availability

Not applicable.

## Abbreviations

AAN: American Academy of Neurology
CRS-r: Coma Recovery Scale—Revised
DOC: Disorders of consciousness
DTI: Diffusion tensor imaging
Dx: Diagnosis
EAN: European Academy of Neurology
EEG: Electroencephalogram
fMRI: Functional magnetic resonance imaging
GRADE: Grading of Recommendations Assessment, Development and Evaluation
MCA: Mental capacity act
MCS: Minimally conscious state
NHS: National Health System
NSF: National Service Framework
PDOC: Prolonged disorders of consciousness
PET: Positron emission tomography
PVS: Persistent vegetative state
Px: Prognosis
RCP: Royal College of Physicians
Sep: Somatosensory evoked potential
SPECT: Single-photon emission computerized tomography
TMS: Transcranial magnetic stimulation
UWS: Unresponsive wakefulness syndrome
VS: Vegetative state
