# Supplementary material for: The importance of instrumental assessment in disorders of consciousness: a comparison between American, European, and UK International recommendations
Source: Crit Care. 2022 Aug 10;26:245. doi: 10.1186/s13054-022-04119-5 (PMC9367125; doi:10.1186/s13054-022-04119-5)
Supplement: Supplementary file 1 — Additional file 1: Comparison between American and European Academies of Neurology eligible studies. [file 13054_2022_4119_MOESM1_ESM.doc]

**Supplementary Materials**

**The importance of instrumental assessment in disorders of consciousness: A**

**comparison between American, European, and UK International recommendations**

**Authors**

Magnani F.G.^1°^, Barbadoro F. ^1°^, Cacciatore M. ^1*^, Leonardi M. ^1^

**Affiliations**

^1^ UOC Neurologia, Salute Pubblica Disabilità – Coma Research Centre - Fondazione IRCCS Istituto Neurologico Carlo Besta; Milan; Italy

°These authors contributed equally to this work

^*^Correspondence to [martina.cacciatore@istituto-besta.it](mailto:martina.cacciatore@istituto-besta.it)

**Comparison between American and European Academies of Neurology eligible studies**

The American Academy of Neurology (AAN) recommendations concerning the use of instrumental tools in the clinical routine with DOC patients ground on 23 reviewed studies, whereas the European Academy of Neurology recommendations are based on 66 reviewed studies (around three times the studies included by the AAN). Moreover, it is worth noting that the 66 studies included by the EAN concerned only the use of instrumental tools for diagnosis, whilst the 23 studies included by the AAN concerned the use of instrumental tools for either the diagnosis or the prognosis, thus further increasing the gap between the two guidelines.

When comparing the studies included in the two guidelines, among the 66 studies retrieved by the EAN, only 8 [1–8] have been included also by the AAN suggesting that this difference depends on the adoption of different inclusion criteria. Thus, we checked whether the unshared studies by the two guidelines conform to this hypothesis.

We started checking whether the above-mentioned studies have not been retrieved due to date constraints implemented into the search strategy: Only one study [9] excluded by the EAN and 9 studies [10–18] excluded by the AAN were justified by this exclusion criterion. The exclusion of the remaining 13 studies by the EAN was explained by different reasons: Two studies [19,20] did not present data at the single-subject level, seven studies [21–27] concerned prognostication without employing consciousness paradigms, three studies [28–30] concerned the use of instrumental tools (i.e., sniff-controller and electromyography) not included in the search strategy, while the exclusion of one study [31] was not justified by any of the EAN exclusion criteria. The exclusion of 48 studies by the AAN (those not justified by the dates constraint) was justified by two main reasons: Twenty-seven studies [32–58] included a sample size <20, and fifteen studies [59–73] included also patients with a time from acute event <28 days. Finally, the remaining seven studies excluded by the AAN [74–80] apparently were not explained by any of the exclusion criteria.

**References**

1. Chennu S, Finoia P, Kamau E, Monti MM, Allanson J, Pickard JD, et al. Dissociable endogenous and exogenous attention in disorders of consciousness. NeuroImage Clin [Internet]. Neuroimage Clin; 2013 [cited 2022 Mar 10];3:450–61. Available from: https://pubmed.ncbi.nlm.nih.gov/24273727/

2. Forgacs PB, Conte MM, Fridman EA, Voss PhD HU, Victor JD, Schiff ND. Preservation of electroencephalographic organization in patients with impaired consciousness and imaging-based evidence of command-following. Ann Neurol [Internet]. Ann Neurol; 2014 [cited 2022 Mar 10];76:869–79. Available from: https://pubmed.ncbi.nlm.nih.gov/25270034/

3. Estraneo A, Loreto V, Guarino I, Boemia V, Paone G, Moretta P, et al. Standard EEG in diagnostic process of prolonged disorders of consciousness. Clin Neurophysiol [Internet]. Clin Neurophysiol; 2016 [cited 2022 Mar 10];127:2379–85. Available from: https://pubmed.ncbi.nlm.nih.gov/27178856/

4. Monti MM, Rosenberg M, Finoia P, Kamau E, Pickard JD, Owen AM. Thalamo-frontal connectivity mediates top-down cognitive functions in disorders of consciousness. Neurology [Internet]. Neurology; 2015 [cited 2022 Mar 10];84:167–73. Available from: https://pubmed.ncbi.nlm.nih.gov/25480912/

5. Kotchoubey B, Lang S, Mezger G, Schmalohr D, Schneck M, Semmler A, et al. Information processing in severe disorders of consciousness: vegetative state and minimally conscious state. Clin Neurophysiol [Internet]. Clin Neurophysiol; 2005 [cited 2022 Mar 10];116:2441–53. Available from: https://pubmed.ncbi.nlm.nih.gov/16002333/

6. Rosazza C, Andronache A, Sattin D, Bruzzone MG, Marotta G, Nigri A, et al. Multimodal study of default-mode network integrity in disorders of consciousness. Ann Neurol [Internet]. Ann Neurol; 2016 [cited 2022 Mar 10];79:841–53. Available from: https://pubmed.ncbi.nlm.nih.gov/26970235/

7. Wang F, Di H, Hu X, Jing S, Thibaut A, Di Perri C, et al. Cerebral response to subject’s own name showed high prognostic value in traumatic vegetative state. BMC Med [Internet]. BMC Med; 2015 [cited 2022 Mar 10];13. Available from: https://pubmed.ncbi.nlm.nih.gov/25880206/

8. Vogel D, Markl A, Yu T, Kotchoubey B, Lang S, Müller F. Can mental imagery functional magnetic resonance imaging predict recovery in patients with disorders of consciousness? Arch Phys Med Rehabil [Internet]. Arch Phys Med Rehabil; 2013 [cited 2022 Mar 10];94:1891–8. Available from: https://pubmed.ncbi.nlm.nih.gov/23735520/

9. Goodwin L. Use of MRI in prediction of recovery from persistent vegetative state - PubMed. J Insur Med [Internet]. 1998 [cited 2022 Mar 9];30:113–4. Available from: https://pubmed.ncbi.nlm.nih.gov/10339293/

10. Chennu S, Annen J, Wannez S, Thibaut A, Chatelle C, Cassol H, et al. Brain networks predict metabolism, diagnosis and prognosis at the bedside in disorders of consciousness. Brain [Internet]. Brain; 2017 [cited 2022 Mar 10];140:2120–32. Available from: https://pubmed.ncbi.nlm.nih.gov/28666351/

11. Curley WH, Forgacs PB, Voss HU, Conte MM, Schiff ND. Characterization of EEG signals revealing covert cognition in the injured brain. Brain [Internet]. Brain; 2018 [cited 2022 Mar 10];141:1404–21. Available from: https://pubmed.ncbi.nlm.nih.gov/29562312/

12. Pavlov YG, Gais S, Müller F, Schönauer M, Schäpers B, Born J, et al. Night sleep in patients with vegetative state. J Sleep Res [Internet]. J Sleep Res; 2017 [cited 2022 Mar 10];26:629–40. Available from: https://pubmed.ncbi.nlm.nih.gov/28444788/

13. Nigri A, Catricalà E, Ferraro S, Bruzzone MG, D’Incerti L, Sattin D, et al. The neural correlates of lexical processing in disorders of consciousness. Brain Imaging Behav [Internet]. Brain Imaging Behav; 2017 [cited 2022 Mar 10];11:1526–37. Available from: https://pubmed.ncbi.nlm.nih.gov/27738996/

14. Braiman C, Fridman EA, Conte MM, Voss HU, Reichenbach CS, Reichenbach T, et al. Cortical Response to the Natural Speech Envelope Correlates with Neuroimaging Evidence of Cognition in Severe Brain Injury. Curr Biol [Internet]. Curr Biol; 2018 [cited 2022 Mar 10];28:3833-3839.e3. Available from: https://pubmed.ncbi.nlm.nih.gov/30471997/

15. Haugg A, Cusack R, Gonzalez-Lara LE, Sorger B, Owen AM, Naci L. Do Patients Thought to Lack Consciousness Retain the Capacity for Internal as Well as External Awareness? Front Neurol [Internet]. Front Neurol; 2018 [cited 2022 Mar 10];9. Available from: https://pubmed.ncbi.nlm.nih.gov/29997565/

16. Kondziella D, Fisher PM, Larsen VA, Hauerberg J, Fabricius M, Møller K, et al. Functional MRI for Assessment of the Default Mode Network in Acute Brain Injury. Neurocrit Care [Internet]. Neurocrit Care; 2017 [cited 2022 Mar 10];27:401–6. Available from: https://pubmed.ncbi.nlm.nih.gov/28484929/

17. Annen J, Blandiaux S, Lejeune N, Bahri MA, Thibaut A, Cho W, et al. BCI Performance and Brain Metabolism Profile in Severely Brain-Injured Patients Without Response to Command at Bedside. Front Neurosci [Internet]. Front Neurosci; 2018 [cited 2022 Mar 10];12. Available from: https://pubmed.ncbi.nlm.nih.gov/29910708/

18. Bodart O, Fecchio M, Massimini M, Wannez S, Virgillito A, Casarotto S, et al. Meditation-induced modulation of brain response to transcranial magnetic stimulation. Brain Stimul [Internet]. Brain Stimul; 2018 [cited 2022 Mar 10];11:1397–400. Available from: https://pubmed.ncbi.nlm.nih.gov/30205950/

19. Zheng XJ, Chen MT, Li JQ, Cao F. Prognosis in prolonged coma patients with diffuse axonal injury assessed by somatosensory evoked potentia. Neural Regen Res [Internet]. Neural Regen Res; 2013 [cited 2022 Mar 9];8:948–54. Available from: https://pubmed.ncbi.nlm.nih.gov/25206387/

20. Estraneo A, Moretta P, Loreto V, Lanzillo B, Cozzolino A, Saltalamacchia A, et al. Predictors of recovery of responsiveness in prolonged anoxic vegetative state. Neurology. 2013;80:464–70.

21. Gosseries O, Schnakers C, Ledoux D, Vanhaudenhuyse A, Bruno MA, Demertzi A, et al. Automated EEG entropy measurements in coma, vegetative state/unresponsive wakefulness syndrome and minimally conscious state. Funct Neurol [Internet]. Funct Neurol; 2011 [cited 2022 Mar 9];26:25–30. Available from: https://pubmed.ncbi.nlm.nih.gov/21693085/

22. Sarà M, Pistoia F, Pasqualetti P, Sebastiano F, Onorati P, Rossini PM. Functional isolation within the cerebral cortex in the vegetative state: a nonlinear method to predict clinical outcomes. Neurorehabil Neural Repair [Internet]. Neurorehabil Neural Repair; 2011 [cited 2022 Mar 9];25:35–42. Available from: https://pubmed.ncbi.nlm.nih.gov/20952634/

23. Pascarella A, Trojano L, Loreto V, Bilo L, Moretta P, Estraneo A. Long-term outcome of patients with disorders of consciousness with and without epileptiform activity and seizures: a prospective single centre cohort study. J Neurol [Internet]. J Neurol; 2016 [cited 2022 Mar 9];263:2048–56. Available from: https://pubmed.ncbi.nlm.nih.gov/27416857/

24. Naro A, Russo M, Leo A, Rifici C, Pollicino P, Bramanti P, et al. Cortical Responsiveness to Nociceptive Stimuli in Patients with Chronic Disorders of Consciousness: Do C-Fiber Laser Evoked Potentials Have a Role? PLoS One [Internet]. PLoS One; 2015 [cited 2022 Mar 9];10. Available from: https://pubmed.ncbi.nlm.nih.gov/26674634/

25. Nayak P, Mahapatra A. Single photon emission computed tomography scanning: A predictor of outcome in vegetative state of head injury. J Neurosci Rural Pract [Internet]. J Neurosci Rural Pract; 2011 [cited 2022 Mar 9];2:12–6. Available from: https://pubmed.ncbi.nlm.nih.gov/21716866/

26. Cavinato M, Freo U, Ori C, Zorzi M, Tonin P, Piccione F, et al. Post-acute P300 predicts recovery of consciousness from traumatic vegetative state. Brain Inj [Internet]. Brain Inj; 2009 [cited 2022 Mar 9];23:973–80. Available from: https://pubmed.ncbi.nlm.nih.gov/19831494/

27. Luauté J, Maucort-Boulch D, Tell L, Quelard F, Sarraf T, Iwaz J, et al. Long-term outcomes of chronic minimally conscious and vegetative states. Neurology [Internet]. Neurology; 2010 [cited 2022 Mar 9];75:246–52. Available from: https://pubmed.ncbi.nlm.nih.gov/20554940/

28. Charland-Verville V, Lesenfants D, Sela L, Noirhomme Q, Ziegler E, Chatelle C, et al. Detection of response to command using voluntary control of breathing in disorders of consciousness. Front Hum Neurosci [Internet]. Front Hum Neurosci; 2014 [cited 2022 Mar 9];8. Available from: https://pubmed.ncbi.nlm.nih.gov/25566035/

29. Habbal D, Gosseries O, Noirhomme Q, Renaux J, Lesenfants D, Bekinschtein TA, et al. Volitional electromyographic responses in disorders of consciousness. Brain Inj [Internet]. Brain Inj; 2014 [cited 2022 Mar 9];28:1171–9. Available from: https://pubmed.ncbi.nlm.nih.gov/24911192/

30. Lesenfants D, Habbal D, Chatelle C, Schnakers C, Laureys S, Noirhomme Q. Electromyographic decoding of response to command in disorders of consciousness. Neurology [Internet]. Neurology; 2016 [cited 2022 Mar 9];87:2099–107. Available from: https://pubmed.ncbi.nlm.nih.gov/27770069/

31. Kotchoubey B, Yu T, Mueller F, Vogel D, Veser S LS. True or false? Activations of language-related areas in patients with disorders of consciousness - PubMed. Curr Pharm Des [Internet]. 2014 [cited 2022 Mar 9];20:4239–47. Available from: https://pubmed.ncbi.nlm.nih.gov/24025064/

32. Landsness E, Bruno MA, Noirhomme Q, Riedner B, Gosseries O, Schnakers C, et al. Electrophysiological correlates of behavioural changes in vigilance in vegetative state and minimally conscious state. Brain [Internet]. Brain; 2011 [cited 2022 Mar 9];134:2222–32. Available from: https://pubmed.ncbi.nlm.nih.gov/21841201/

33. Rosanova M, Gosseries O, Casarotto S, Boly M, Casali AG, Bruno MA, et al. Recovery of cortical effective connectivity and recovery of consciousness in vegetative patients. Brain [Internet]. Brain; 2012 [cited 2022 Mar 10];135:1308–20. Available from: https://pubmed.ncbi.nlm.nih.gov/22226806/

34. Fernandez-Espejo D, Junque C, Vendrell P, Bernabeu M, Roig T, Bargallo N, et al. Cerebral response to speech in vegetative and minimally conscious states after traumatic brain injury. Brain Inj [Internet]. Brain Inj; 2008 [cited 2022 Mar 10];22:882–90. Available from: https://pubmed.ncbi.nlm.nih.gov/18850346/

35. Heelmann V, Lippert-Grüner M, Rommel T, Wedekind C. Abnormal functional MRI BOLD contrast in the vegetative state after severe traumatic brain injury. Int J Rehabil Res [Internet]. Int J Rehabil Res; 2010 [cited 2022 Mar 10];33:151–7. Available from: https://pubmed.ncbi.nlm.nih.gov/19966571/

36. Kremer S, Nicolas-Ong C, Schunck T, Schenck M, Collange O, Mutschler V, et al. Usefulness of functional MRI associated with PET scan and evoked potentials in the evaluation of brain functions after severe brain injury: preliminary results. J Neuroradiol = J Neuroradiol [Internet]. J Neuroradiol; 2010 [cited 2022 Mar 10];37:159–66. Available from: https://pubmed.ncbi.nlm.nih.gov/19781782/

37. Okumura Y, Asano Y, Takenaka S, Fukuyama S, Yonezawa S, Kasuya Y, et al. Brain activation by music in patients in a vegetative or minimally conscious state following diffuse brain injury. Brain Inj [Internet]. Brain Inj; 2014 [cited 2022 Mar 10];28:944–50. Available from: https://pubmed.ncbi.nlm.nih.gov/24655034/

38. Qin P, Di H, Liu Y, Yu S, Gong Q, Duncan N, et al. Anterior cingulate activity and the self in disorders of consciousness. Hum Brain Mapp [Internet]. Hum Brain Mapp; 2010 [cited 2022 Mar 10];31:1993–2002. Available from: https://pubmed.ncbi.nlm.nih.gov/20336686/

39. Sharon H, Pasternak Y, Ben Simon E, Gruberger M, Giladi N, Krimchanski BZ, et al. Emotional processing of personally familiar faces in the vegetative state. PLoS One [Internet]. PLoS One; 2013 [cited 2022 Mar 10];8. Available from: https://pubmed.ncbi.nlm.nih.gov/24086365/

40. Bekinschtein TA, Dehaene S, Rohaut B, Tadel F, Cohen L, Naccache L. Neural signature of the conscious processing of auditory regularities. Proc Natl Acad Sci U S A [Internet]. Proc Natl Acad Sci U S A; 2009 [cited 2022 Mar 10];106:1672–7. Available from: https://pubmed.ncbi.nlm.nih.gov/19164526/

41. Bardin JC, Fins JJ, Katz DI, Hersh J, Heier LA, Tabelow K, et al. Dissociations between behavioural and functional magnetic resonance imaging-based evaluations of cognitive function after brain injury. Brain [Internet]. Brain; 2011 [cited 2022 Mar 10];134:769–82. Available from: https://pubmed.ncbi.nlm.nih.gov/21354974/

42. Bardin JC, Schiff ND, Voss HU. Pattern classification of volitional functional magnetic resonance imaging responses in patients with severe brain injury. Arch Neurol [Internet]. Arch Neurol; 2012 [cited 2022 Mar 10];69:176–81. Available from: https://pubmed.ncbi.nlm.nih.gov/22332186/

43. Bick AS, Leker RR, Ben-Hur T, Levin N. Implementing novel imaging methods for improved diagnosis of disorder of consciousness patients. J Neurol Sci [Internet]. J Neurol Sci; 2013 [cited 2022 Mar 10];334:130–8. Available from: https://pubmed.ncbi.nlm.nih.gov/24007872/

44. Cruse D, Chennu S, Chatelle C, Bekinschtein TA, Fernández-Espejo D, Pickard JD, et al. Bedside detection of awareness in the vegetative state: a cohort study. Lancet (London, England) [Internet]. Lancet; 2011 [cited 2022 Mar 10];378:2088–94. Available from: https://pubmed.ncbi.nlm.nih.gov/22078855/

45. Gibson RM, Chennu S, Fernández-Espejo D, Naci L, Owen AM, Cruse D. Somatosensory attention identifies both overt and covert awareness in disorders of consciousness. Ann Neurol [Internet]. Ann Neurol; 2016 [cited 2022 Mar 10];80:412–23. Available from: https://pubmed.ncbi.nlm.nih.gov/27422169/

46. Gibson RM, Fernandez-Espejo D, Gonzalez-Lara LE, Kwan BY, Lee DH, Owen AM, et al. Multiple tasks and neuroimaging modalities increase the likelihood of detecting covert awareness in patients with disorders of consciousness. Front Hum Neurosci [Internet]. Front Hum Neurosci; 2014 [cited 2022 Mar 10];8. Available from: https://pubmed.ncbi.nlm.nih.gov/25505400/

47. Rodriguez Moreno D, Schiff ND, Giacino J, Kalmar K, Hirsch J. A network approach to assessing cognition in disorders of consciousness. Neurology [Internet]. Neurology; 2010 [cited 2022 Mar 10];75:1871–8. Available from: https://pubmed.ncbi.nlm.nih.gov/20980667/

48. Soddu A, Vanhaudenhuyse A, Bahri MA, Bruno MA, Boly M, Demertzi A, et al. Identifying the default-mode component in spatial IC analyses of patients with disorders of consciousness. Hum Brain Mapp [Internet]. Hum Brain Mapp; 2012 [cited 2022 Mar 10];33:778–96. Available from: https://pubmed.ncbi.nlm.nih.gov/21484953/

49. Bodart O, Gosseries O, Wannez S, Thibaut A, Annen J, Boly M, et al. Measures of metabolism and complexity in the brain of patients with disorders of consciousness. NeuroImage Clin [Internet]. Neuroimage Clin; 2017 [cited 2022 Mar 9];14:354–62. Available from: https://pubmed.ncbi.nlm.nih.gov/28239544/

50. Casali AG, Gosseries O, Rosanova M, Boly M, Sarasso S, Casali KR, et al. A theoretically based index of consciousness independent of sensory processing and behavior. Sci Transl Med [Internet]. Sci Transl Med; 2013 [cited 2022 Mar 10];5. Available from: https://pubmed.ncbi.nlm.nih.gov/23946194/

51. Ragazzoni A, Pirulli C, Veniero D, Feurra M, Cincotta M, Giovannelli F, et al. Vegetative versus minimally conscious states: a study using TMS-EEG, sensory and event-related potentials. PLoS One [Internet]. PLoS One; 2013 [cited 2022 Mar 10];8. Available from: https://pubmed.ncbi.nlm.nih.gov/23460826/

52. Sergent C, Faugeras F, Rohaut B, Perrin F, Valente M, Tallon-Baudry C, et al. Multidimensional cognitive evaluation of patients with disorders of consciousness using EEG: A proof of concept study. NeuroImage Clin [Internet]. Neuroimage Clin; 2016 [cited 2022 Mar 10];13:455–69. Available from: https://pubmed.ncbi.nlm.nih.gov/28116238/

53. Lulé D, Noirhomme Q, Kleih SC, Chatelle C, Halder S, Demertzi A, et al. Probing command following in patients with disorders of consciousness using a brain-computer interface. Clin Neurophysiol [Internet]. Clin Neurophysiol; 2013 [cited 2022 Mar 10];124:101–6. Available from: https://pubmed.ncbi.nlm.nih.gov/22920562/

54. De Tommaso M, Navarro J, Ricci K, Lorenzo M, Lanzillotti C, Colonna F, et al. Pain in prolonged disorders of consciousness: laser evoked potentials findings in patients with vegetative and minimally conscious states. Brain Inj [Internet]. Brain Inj; 2013 [cited 2022 Mar 10];27:962–72. Available from: https://pubmed.ncbi.nlm.nih.gov/23789870/

55. Risetti M, Formisano R, Toppi J, Quitadamo LR, Bianchi L, Astolfi L, et al. On ERPs detection in disorders of consciousness rehabilitation. Front Hum Neurosci [Internet]. Front Hum Neurosci; 2013 [cited 2022 Mar 10];7. Available from: https://pubmed.ncbi.nlm.nih.gov/24312041/

56. Perrin F, Schnakers C, Schabus M, Degueldre C, Goldman S, Brédart S, et al. Brain response to one’s own name in vegetative state, minimally conscious state, and locked-in syndrome. Arch Neurol [Internet]. Arch Neurol; 2006 [cited 2022 Mar 10];63:562–9. Available from: https://pubmed.ncbi.nlm.nih.gov/16606770/

57. Bekinschtein TA, Manes FF, Villarreal M, Owen AM, Della-Maggiore V. Functional imaging reveals movement preparatory activity in the vegetative state. Front Hum Neurosci [Internet]. Front Hum Neurosci; 2011 [cited 2022 Mar 10];5:1–11. Available from: https://pubmed.ncbi.nlm.nih.gov/21441977/

58. Coleman MR, Rodd JM, Davis MH, Johnsrude IS, Menon DK, Pickard JD, et al. Do vegetative patients retain aspects of language comprehension? Evidence from fMRI. Brain [Internet]. Brain; 2007 [cited 2022 Mar 10];130:2494–507. Available from: https://pubmed.ncbi.nlm.nih.gov/17827174/

59. Schnakers C, Vanhaudenhuyse A, Giacino J, Ventura M, Boly M, Majerus S, et al. Diagnostic accuracy of the vegetative and minimally conscious state: clinical consensus versus standardized neurobehavioral assessment. BMC Neurol [Internet]. BMC Neurol; 2009 [cited 2022 Mar 9];9. Available from: https://pubmed.ncbi.nlm.nih.gov/19622138/

60. Sitt JD, King JR, El Karoui I, Rohaut B, Faugeras F, Gramfort A, et al. Large scale screening of neural signatures of consciousness in patients in a vegetative or minimally conscious state. Brain [Internet]. Brain; 2014 [cited 2022 Mar 10];137:2258–70. Available from: https://pubmed.ncbi.nlm.nih.gov/24919971/

61. Monti MM, Vanhaudenhuyse A, Coleman MR, Boly M, Pickard JD, Tshibanda L, et al. Willful modulation of brain activity in disorders of consciousness. N Engl J Med [Internet]. N Engl J Med; 2010 [cited 2022 Mar 10];362:579–89. Available from: https://pubmed.ncbi.nlm.nih.gov/20130250/

62. Demertzi A, Antonopoulos G, Heine L, Voss HU, Crone JS, De Los Angeles C, et al. Intrinsic functional connectivity differentiates minimally conscious from unresponsive patients. Brain [Internet]. Brain; 2015 [cited 2022 Mar 10];138:2619–31. Available from: https://pubmed.ncbi.nlm.nih.gov/26117367/

63. Demertzi A, Gómez F, Crone JS, Vanhaudenhuyse A, Tshibanda L, Noirhomme Q, et al. Multiple fMRI system-level baseline connectivity is disrupted in patients with consciousness alterations. Cortex [Internet]. Cortex; 2014 [cited 2022 Mar 10];52:35–46. Available from: https://pubmed.ncbi.nlm.nih.gov/24480455/

64. Crone JS, Schurz M, Höller Y, Bergmann J, Monti M, Schmid E, et al. Impaired consciousness is linked to changes in effective connectivity of the posterior cingulate cortex within the default mode network. Neuroimage [Internet]. Neuroimage; 2015 [cited 2022 Mar 10];110:101–9. Available from: https://pubmed.ncbi.nlm.nih.gov/25620493/

65. Stender J, Mortensen KNN, Thibaut A, Darkner S, Laureys S, Gjedde A, et al. The Minimal Energetic Requirement of Sustained Awareness after Brain Injury. Curr Biol [Internet]. Curr Biol; 2016 [cited 2022 Mar 10];26:1494–9. Available from: https://pubmed.ncbi.nlm.nih.gov/27238279/

66. Malinowska U, Chatelle C, Bruno MA, Noirhomme Q, Laureys S, Durka PJ. Electroencephalographic profiles for differentiation of disorders of consciousness. Biomed Eng Online [Internet]. Biomed Eng Online; 2013 [cited 2022 Mar 10];12. Available from: https://pubmed.ncbi.nlm.nih.gov/24143892/

67. Faugeras F, Rohaut B, Weiss N, Bekinschtein T, Galanaud D, Puybasset L, et al. Event related potentials elicited by violations of auditory regularities in patients with impaired consciousness. Neuropsychologia [Internet]. Neuropsychologia; 2012 [cited 2022 Mar 10];50:403–18. Available from: https://pubmed.ncbi.nlm.nih.gov/22230230/

68. Faugeras F, Rohaut B, Weiss N, Bekinschtein TA, Galanaud D, Puybasset L, et al. Probing consciousness with event-related potentials in the vegetative state. Neurology [Internet]. Neurology; 2011 [cited 2022 Mar 10];77:264–8. Available from: https://pubmed.ncbi.nlm.nih.gov/21593438/

69. Rohaut B, Faugeras F, Chausson N, King JR, Karoui I El, Cohen L, et al. Probing ERP correlates of verbal semantic processing in patients with impaired consciousness. Neuropsychologia [Internet]. Neuropsychologia; 2015 [cited 2022 Mar 10];66:279–92. Available from: https://pubmed.ncbi.nlm.nih.gov/25447058/

70. Steppacher I, Eickhoff S, Jordanov T, Kaps M, Witzke W, Kissler J. N400 predicts recovery from disorders of consciousness. Ann Neurol [Internet]. Ann Neurol; 2013 [cited 2022 Mar 10];73:594–602. Available from: https://pubmed.ncbi.nlm.nih.gov/23443907/

71. Crone JS, Ladurner G, Höller Y, Golaszewski S, Trinka E, Kronbichler M. Deactivation of the default mode network as a marker of impaired consciousness: an fMRI study. PLoS One [Internet]. PLoS One; 2011 [cited 2022 Mar 10];6. Available from: https://pubmed.ncbi.nlm.nih.gov/22039473/

72. Li L, Kang X gang, Qi S, Xu X xia, Xiong L ze, Zhao G, et al. Brain response to thermal stimulation predicts outcome of patients with chronic disorders of consciousness. Clin Neurophysiol [Internet]. Clin Neurophysiol; 2015 [cited 2022 Mar 10];126:1539–47. Available from: https://pubmed.ncbi.nlm.nih.gov/25468244/

73. Stender J, Gosseries O, Bruno MA, Charland-Verville V, Vanhaudenhuyse A, Demertzi A, et al. Diagnostic precision of PET imaging and functional MRI in disorders of consciousness: a clinical validation study. Lancet (London, England) [Internet]. Lancet; 2014 [cited 2022 Mar 10];384:514–22. Available from: https://pubmed.ncbi.nlm.nih.gov/24746174/

74. Cruse D, Chennu S, Chatelle C, Fernández-Espejo D, Bekinschtein TA, Pickard JD, et al. Relationship between etiology and covert cognition in the minimally conscious state. Neurology. 2012;78:816–22.

75. Boly M, Garrido MI, Gosseries O, Bruno MA, Boveroux P, Schnakers C, et al. Preserved feedforward but impaired top-down processes in the vegetative state. Science (80- ). 2011;332:858–62.

76. Fischer C, Luaute J, Morlet D. Event-related potentials (MMN and novelty P3) in permanent vegetative or minimally conscious states. Clin Neurophysiol. 2010;121:1032–42.

77. Cologan V, Drouot X, Parapatics S, Delorme A, Gruber G, Moonen G, et al. Sleep in the unresponsive wakefulness syndrome and minimally conscious state. J Neurotrauma. 2013;30:339–46.

78. De Biase S, Gigli GL, Lorenzut S, Bianconi C, Sfreddo P, Rossato G, et al. The importance of polysomnography in the evaluation of prolonged disorders of consciousness: Sleep recordings more adequately correlate than stimulus-related evoked potentials with patients’ clinical status. Sleep Med. 2014;15:393–400.

79. Coleman MR, Davis MH, Rodd JM, Robson T, Ali A, Owen AM, et al. Towards the routine use of brain imaging to aid the clinical diagnosis of disorders of consciousness. Brain. 2009;132:2541–52.

80. Nigri A, Ferraro S, Bruzzone MG, Nava S, D’Incerti L, Bertolino N, et al. Central olfactory processing in patients with disorders of consciousness. Eur J Neurol. 2016;23:605–12.
